# How Physical and Molecular Anthropology Interplay in the Creation of Biological Profiles of Unidentified Migrants

**DOI:** 10.3390/genes14030706

**Published:** 2023-03-13

**Authors:** Elena Pilli, Andrea Palamenghi, Stefania Morelli, Debora Mazzarelli, Danilo De Angelis, Richard L. Jantz, Cristina Cattaneo

**Affiliations:** 1 Laboratorio di Antropologia Molecolare Forense, Dipartimento di Biologia, Università degli Studi di Firenze, Via del Proconsolo 12, 50122 Florence, Italy; 2 LABANOF, Laboratorio di Antropologia e Odontologia Forense, Dipartimento di Scienze Biomediche per la Salute, Università degli Studi di Milano, Via L. Mangiagalli 37, 20133 Milan, Italy; 3 Department of Anthropology, University of Tennessee, 505 Strong Hall, Knoxville, TN 37996-0720, USA

**Keywords:** forensic anthropology, DNA, skeletal sex, ancestry, migrants

## Abstract

The skeletal sex and ancestry of unidentified human crania can be inferred both from physical and from molecular features. This paper depicts and discusses the experiences of physical and molecular anthropologists on a set of commingled crania from the largest Mediterranean shipwreck disaster on 18 April 2015, in order to facilitate identification of human crania. Twenty-one disarticulated crania that were recovered from the above-mentioned shipwreck were analyzed to estimate skeletal sex and ancestry, following a physical and a molecular pipeline. The physical analyses applied morphological and metric methods that provided posterior probabilities for the crania to be classified into a sex or ancestral group. The molecular analyses were performed on petrous bones via a shotgun sequencing approach that allowed us to determine the sex of each individual and to retrieve the complete mitochondrial genome, Y chromosome single nucleotide polymorphisms, up to 597573 SNPs across the human genome from each individual. The morphometric sex analyses showed that most crania belonged to male individuals, although some estimations remained uncertain or undetermined. Inconsistent results were obtained for ancestry estimation as well, since morphological methods classified the crania mostly as European/White, in contrast to the most numerous African forms determined by craniometric analyses. This quite agreed with molecular analyses that identified only African males. Overall, undetermined and contrasting results were obtained between disciplines, preventing the creation of reliable and sound biological profiles that could provide guidance on the sex and ancestral group of the victims. Therefore, the times may not be mature for a merger of physical and molecular anthropology. However, future investigations of this research avenue would pave the way to the possible development of novel tools, methods, and wider reference databases that could address the limitations of both disciplines.

## 1. Introduction

Recently, humanitarian emergencies related to forced migrations have intensified, and thus, 82.4 million people are estimated to be forcibly displaced worldwide [1]. Unfortunately, migration journeys and attempts at crossing borders have often resulted in mass fatalities. In Europe, this phenomenon has consistently increased since 2013, especially in the Mediterranean Sea, with countless tragedies that, most times, have remained silent and unreported [2]. Since 2014, the Mediterranean route has accounted for at least 25,000 migrant deaths [3], and therefore, it is considered to be the deadliest path from African and Eastern countries towards Europe. Within this plight, the victims’ rights to identity and the relatives’ rights to know should always be granted [4]. Therefore, forensic investigators have been requested to give a name to a victim, and, as part of their procedure, to first determine their biological profile that will provide insightful information to be compared against missing persons’ data [5]. To do so, forensic experts must begin, particularly when working on skeletal remains, with estimations of biological sex, ancestry, age at death, and stature.

Skeletal sex and ancestry can be inferred both from anthropological evidence and from molecular evidence. Traditionally, physical anthropologists have deemed the human cranium as a reliable indicator for sex and ancestry estimation of unknown remains. On the basis of morphology and metrics, the cranium presents dimorphic traits between sexes [6,7] and it has also been demonstrated that variations of morphological cranial features exist between populations, according to the ancestral groups [8,9]. To this end, qualitative and quantitative methods have been developed that include morphological observations and metric analyses. Starting from the work of Buikstra and Ubelaker [10], Walker [7] proposed a scoring system of cranial traits that included the nuchal crest, mastoid process, supraorbital margin, glabella, and mental eminence and developed sex determination discriminant functions to determine the biological sex. This was the first study to provide a statistical framework for a subjective evaluation of cranial morphological traits for sex estimation. However, the author suggested caution in using the functions on populations other than the reference. The issue of population-specific equations was addressed by Krüger et al. [11]. The authors reported low accuracies of Walker’s method when the discriminant functions were applied to a South African sample and provided modified equations. Similarly, Cappella et al. [12] developed specific regression models for an Italian contemporary cemetery population. The above-mentioned issues highlight that the morphological sex estimation method has some weak points when performed on unknown samples. Craniometric analysis represents a reliable mean for biological sex and ancestry estimation, although the lack of reference samples from which the statistical framework is inferred could complicate interpretation of the results [13]. However, in investigations such as the identification of unknown migrants, craniometric data have proven to be valuable indicators for classification into ancestral groups [5].

Since ancestry can be considered to be the genetic inheritance everyone receives from their ancestors and from population members that have occupied the same place of origin for long periods of time, molecular anthropologists could also provide valuable support to infer the biogeographical origin via DNA analysis. Recent advances in molecular technology associated with the possibility to also simultaneously genotype hundreds of markers from degraded bone samples have resulted in accurate and efficient estimation of an individual ancestry that could implement anthropological studies with additional evidence. DNA profiling has played an essential role in the positive identification of victims of several humanitarian cases, including mass burials in Guatemala [14,15] and Bosnia-Herzegovina [15], and mass disasters such as the World Trade Center, the Indonesian Tsunami [16,17], and the massacre at Fosse Ardeatine in Italy [18] (where reference DNA profiles from family members are available for comparison with those of the victims) but this is largely different from constructing a biological profile when there is no hypothesis of identity. From this perspective, DNA analyses on bones could provide further information to predict, for example, the biogeographical ancestry of a person of interest.

The fusion of physical and genetic anthropological methods, although apparently useful, may create further complications in the interpretation of a biological profile. In this article, we aim to share the experience of the application of both methods on a set of commingled crania belonging to the largest Mediterranean shipwreck disaster on 18 April 2015, in order to facilitate identification of human crania. In the context of the examination of the remains of circa 1000 victims, the present study focused on 21 disarticulated crania of these victims that were analyzed from a physical and genetic perspective in order to infer biological sex and ancestry. The final purpose, along with aiding identification, is to present the challenges that the practitioners faced when working on such a peculiar set of unknown remains and to highlight how forensic anthropologists (physical and molecular) can try to interplay in a conjoined investigation to provide more robust information and how this combination may still need further research for proper integration.

## 2. Materials and Methods

The sample included 21 disarticulated crania that were recovered from the shipwreck of 18 April 2015. The study complies with Police Mortuary Rules (DPR 09.10.1990 No. 285, art. 43) and the *Regio Decreto* (08.31.1933 No. 1592, art. 32) and is encompassed within the identification project of the victims enshrined in an MOU between the University of Milan and the Italian government. The crania were brushed with water and soap to remove dirt and soft tissue on the external surface and within the skull. Morphological and metric analyses were performed prior to sampling for DNA typing. Dental development was investigated through radiographic images performed with a portable dental X-ray unit (intraoral sensor (EZ Sensor 1.5, Vatech, Hwaseong, Republic of Korea) and X-ray tube (model Rextar, Poskom Co. Ltd., Goyang, Republic of Korea)). There were 14 crania with completed dental development (the apices of the third molar completely closed). Seven crania presented evidence of on-going root growth of third molars which were evaluated according to Mincer et al. [19]. Since the reference populations of the method are hardly representative of the ancestry of the sample, only the stage of development is provided (Table 1). For all crania, the petrous bone was chosen as the preferred anatomical element for DNA analysis [20,21,22] and was sampled with a Stryker hacksaw.

### 2.1. Anthropological Methods

#### Morphometric Ancestry and Sex Estimation

The morphological analysis assessed the 11 morphoscopic traits originally suggested by Hefner [9]: anterior nasal spine (ANS), inferior nasal aperture (INA), interorbital breadth (IOB), malar tubercle (MT), nasal aperture width (NAW), nasal bone contour (NBC), nasal overgrowth (NO), postbregmatic depression (PBD), supranasal suture (SPS), transverse palatine suture (TPS), and zygomaticomaxillary suture (ZS). The optimized summed scored attributes (OSSA) scores were calculated based on the scores attributed to the ANS, INA, IOB, NAW, NBC, and PBD [23]. This method considers two major classification groups, i.e., Black and White. In addition, the decision support system, HefneR on the web-based platform Osteomics [24] was used to include additional groups (African, American Indian, Asian, and European).

**Table 1:** Summary of the sex and ancestry estimation of the 21 crania. M/F, a cranium with a higher number of probabilities that classify it as male; F/M, more probabilities classify the cranium as female. In brackets is the highest posterior probability for the cranium to be assigned to an ancestral or sex group.

			Ancestry Estimation			Sex Estimation			
ID	Mandible	Tooth Stage [19]	HefneR [24]	OSSA Score [23]	Craniometrics [25]	South African White [11]	South African Black [11]	Walker [7]	Craniometrics [25]
003		G	African (0.85)	Black	West Africa (0.67)	F/M	M	M	M (0.93)
028		F	European (0.87)	White	Somali (0.97)	F/M	F	F/M	F (0.95)
058 A1	X	Adult	European (0.51)	White	Somali (0.58)	F/M	M	M	M (0.72)
062	X	G	African (0.99)	Black	West Africa (0.91)	M	M	M	M (0.82)
095		Adult	African (0.80)	White	Portuguese (0.86)	F	M	M	M (0.77)
099		Adult	European (0.99)	White	West Africa (0.41)	F	M	M	M (0.69)
100-1	X	Adult	European (0.95)	White	Somali (0.96)	F	F	F	F (0.98)
100-2	X	Adult	European (0.96)	White	Hainan (0.41)	M/F	M	M	M (0.97)
104-1		Adult	African (0.98)	Black	West Africa (0.73)	F/M	M	M/F	M/F (0.51)
104-5		F	African (0.59)	Black	Somali (0.92)	F	F	F	F (0.91)
105-2	X	F	African (0.65)	White	West Africa (0.32)	M	M	M	M (0.99)
125		Adult	European (0.47)	White	Somali (0.38)	F/M	M	M	F/M (0.51)
131	X	Adult	European (0.69)	White	Zulu (0.24)	M/F	M/F	M/F	M (0.75)
137	X	Adult	European (0.84)	White	Somali (0.98)	F/M	M	M	M (0.74)
146		Adult	European (0.84)	White	West Africa (0.33)	M	M	M	M (0.93)
149-1	X	G	European (0.99)	White	Euro-American (0.31)	M/F	M/F	M	M (0.99)
154-1	X	F	African (0.95)	Black	Somali (0.48)	F/M	M/F	M	F (0.76)
154-2	X	Adult	African (0.99)	Black	Somali (0.70)	M	M	M	M (0.99)
178-1	X	Adult	Asian (0.34)	Black	Somali (0.42)	M/F	M	M	M (0.75)
178-2		Adult	Asian (0.49)	White	West Africa (0.42)	M	M	M	F (0.99)
178-3		Adult	Asian (0.73)	White	Somali (0.47)	F	F	F	F (0.59)

Metric data were acquired using a microscribe digitizer, and the 3Skull software [25] that obtains the coordinates x, y, and z of each landmark and calculates the inter-landmark distances of the measurements defined by Howells [26]. The crania were classified against West and East African, Somalian, Zulu, Hainan Chinese, Portuguese, and Euro-American population samples. The West African group of population samples are described in detail in Spradley and Jantz [27]. The Somalian group of population samples were likely battlefield casualties of the Italian invasion of British Somiland in 1940. The Zulu and Hainan group of population samples are from Howells [26]. The African group of population samples all date to the 19th or early 20th century. As such, the population samples are not entirely suitable as groups to compare with the modern crania represented by the shipwreck victims that may have experienced secular change. The analysis of the 21 crania was a two-step process. The first step was to estimate the population affinity of each cranium based on its morphometric similarity to a reference sample. Step two was a search for a substructure that may exist among the crania, based on their distances from one another. Step one used the Mahalanobis generalized distance of each of the 21 crania from the reference samples. The principal component scores for the reference samples were derived from within the covariance matrix. Then, the eigenvalues and eigenvectors were used to obtain principal component scores for each of the 21 crania used in the present study. They were used to compute the Mahalanobis distance of each cranium from the reference samples. A cranium’s population affinity was assigned to the group to which it had the lowest Mahalanobis distance. Step two used the Mahalanobis distance of each cranium from every other cranium, yielding a 21 × 21 distance matrix. The principal coordinates from this matrix were extracted using the procedure described by Gower [28]. The principal coordinates were calculated in such a way as to maximize variation among crania variances. It is important to stress that the principal coordinates are chosen without regard to the population affinity described above. The only relationship to the reference sample is that the reference sample within the covariance matrix is used to obtain the principal component scores. This only requires that the covariance matrices from which the 21 crania were drawn do not differ significantly from those of the reference samples, which is not an unreasonable assumption. After obtaining the principal coordinates, post hoc tests of whether they sort populations according to affinities identified in Step one were performed. The population affinity of each cranium was attached to its principal coordinate score. The scores could then be displayed graphically and analyzed statistically.

Skeletal sex estimation was based on the five morphological traits suggested by Walker [7]: nuchal crest, mastoid process, supraorbital margin, glabella, and mental eminence. The five traits were scored for each cranium, except for the mental eminence that could not be evaluated in all cases, since 10 crania did not present the mandible. The skeletal sex was estimated by calculating the equations that were related to the populations indicated by the ancestry estimation. When a cranium was classified as White, Krüger’s equations [11] for White South African were applied, and vice versa when a cranium was classified as Black. In addition, the original equations by Walker [7] were applied, although the method does not provide specific equations for Black and White individuals. A cranium was considered to be male (M) or female (F) when all the traits considered by the equations agreed. When a cranium presented mixed traits, it was not possible to express a definitive judgment. This meant that, in some instances, the highest probability when considering one combination of traits (e.g., glabella and mastoid process) classified the cranium as female and as male when considering another couple of traits (e.g., nuchal crest and mastoid process). As such, the sex estimation is indicated as M/F, if the cranium has a higher number of probabilities that classify it as male, or as F/M if more probabilities classify the cranium as female. The metric data acquired for ancestry estimation were also used for craniometric sex estimation. The Somalian crania were from males only, but all other samples included both sexes. Sexes were assigned from cranial morphology, except for Zulu, which were samples of a known sex. Zulu crania were used to develop sex discriminant functions using three highly dimorphic dimensions, glabellar projection (GLS), bizygomatic breadth (ZYB), and mastoid height (MDH).

### 2.2. Molecular Methods

#### 2.2.1. Sample Preparation, DNA Extraction, Whole-Genome Library Preparation, and Sequencing

Bone samples were processed in the molecular anthropology unit of the University of Florence, a state-of-the-art facility dedicated to the analysis of degraded DNA samples. To remove potential contaminants, the outer layer of petrous bones was brushed using a dentist drill with disposable tips as suggested by Pinhasi et al. [21] and irradiated with UV light (λ  =  254 nm) for 45 min in a Biolink DNA Crosslinker (Biometra, Goettingen, Germany). A minimally invasive approach was followed to recover approximately 50 mg of bone powder from petrous bones, as described by Sirak et al. [29]. DNA was extracted using silica-based protocol [30] and eluted in TET buffer (10 nM Tris, 1 mM EDTA, 0.05% Tween-20) twice for a final volume of 100 μL Subsequently, a double-stranded and dual-indexed Illumina DNA library was prepared from 25 µL of each extract following previously published protocols [31,32]. For all samples, deaminated cytosines resulting from DNA damage were partially removed using an uracil-DNA-glycosylase treatment (UDG-half) as proposed by Rohland et al. [33]. Then, a unique combination of two indices per library was used for barcoding, the libraries were pooled in an equimolar amount and sequenced in 200 bp paired-end mode on a NovaSeq 6000 instrument (Illumina, San Diego, CA, USA) for a depth of ~14 million reads for samples, except for 58-A1, 62, 131, and 149-1 for which deep sequencing was performed. Negative controls were used in all the experimental steps to monitor the absence of contaminants in the reagent and the environment.

#### 2.2.2. Postsequencing Data Processing

Raw data were demultiplexed, allowing for a maximum of one mismatch in each index, and processed through the EAGER pipeline (v1.92.59) [34] keeping paired-end reads separate. Adapters were trimmed using Clip&Merge v1.7.4 with the parameter “perform only adapter clipping” [34] and reads shorter than 30 bp were discarded. Filtered reads were subsequently mapped against the human reference genome (Hg19) using BWA v. 0.7.10 [35] with the stringency parameter set to 0.01 and a mapping quality filter of 30. The duplicate removal was performed using DeDup (v0.12.2) [34], which considered both start and end coordinates of the reads to identify and discard identical sequences.

Before genotype calling, reads mapped onto the human genome were authenticated by deamination and fragmentation pattern analysis using mapDamage2.0 [36]. The presence of degraded DNA typical features was checked as a short average length of DNA sequences and an increased proportion of miscoding lesions at the terminal position of the molecules. Simultaneously, extraction and library preparation blanks were also analyzed for the presence of potential background contamination during the laboratory activity.

#### 2.2.3. Sex Determination

The molecular sex was estimated from each individual by comparing the number of alignments to the Y chromosome and the total number of alignments to the X and Y chromosomes in the libraries prepared [37].

#### 2.2.4. Genotype Calling and Principal Component Analysis for Ancestry Inference

Genotyping was carried out on the BAM files. A pseudo-haploid genotype was reconstructed for each individual with pileupCaller (Phred-scaled base quality score ≥30) by performing a calling of 597,573 alleles from the 1240 K + human origin dataset. Subsequently, genotyped data were merged with the human origin panel and the 1240 K dataset (https://reich.hms.harvard.edu accessed on 16 November 2022) selecting only 7566 modern individuals (African = 1195, European = 1237, Asian = 4563, American = 564, and Ocean = 7). The software smartpca from the EIGENSOFT package (v16000) [38] was used to compute a world PCA using all individuals of the human origin dataset.

#### 2.2.5. Haplogroup Assignment of Uniparental Markers

To perform the mtDNA analysis, reads were processed with EAGER and alignment to the mitochondrial reference genome (rCRS) using CircularMapper, a tool that considers the circularity of the mtDNA [34]. Reads with mapping quality ≥30 were selected and extracted from the BAM files using SAMtools v1.7 [39], and the consensus sequence was called using mpileup and vcfutils.pl in the SAMtools package. The mitochondrial haplogroups were assigned according to the rCRS using MITOMASTER [40,41]. The D-loop variants (16024–576 bp) of each mitochondrial genome obtained from the previous analysis with MITOMASTER were uploaded into the EMPOP platform (v4/R13) (http://empop.online/ accessed on 19 December 2022) to calculate the frequency of the genetic profile within the reference database and to assess its worldwide distribution through heatmaps [42].

The Y chromosome haplogroup was also determined using the Yleaf 2.2 software [43], a Phython-based tool for effective analysis and interpretation of Y chromosome NGS data. This easy-to-use and publicly available software performs NRY single nucleotide polymorphism calling and subsequent haplogroup inference, based on ISOGG markers (https://isogg.org/tree/ accessed on 13 December 2022), starting from the BAM file. Reads with mapping quality ≥30 were selected.

## 3. Results and Discussion

### 3.1. Physical Data

#### Skeletal Ancestry and Sex Estimation

Regarding morphoscopic ancestry, 14 crania were classified as White and 7 crania were classified as Black, according to the OSSA scores [23]. The decision support system HefneR [24] provided the following outcomes based on the highest probability: 10 crania were classified as European, 8 crania were classified as African, and 3 crania were classified as Asian. In detail, 12 crania were classified into a single population group with a probability equal to or above 0.8; in six cases, the highest probability was lower than 0.8 (between 0.72 and 0.50); in three crania, the highest probabilities to be classified in one group were equal to or lower than 0.50, with lower distributions in other classification groups. In five cases, inconsistent results between the two morphoscopic methods were obtained: in these instances, the same cranium was classified into two different population groups according to the OSSA score and HefneR. For example, the cranium Sample 095 was classified as White by the OSSA score, but its highest posterior probability (pp = 0.8) classified it as African by using HefneR.

An OSSA score identifies two major categories, i.e., Whites and Blacks, based on the score assigned to six of the traits suggested by Hefner [9]. Although this binary classification provides indications on the ancestral group of a crania from which one can start the ancestry analysis, it does not suggest further details. In contrast, the decision support system HefneR outputs posterior probabilities according to the affinity of all the traits by Hefner [9] for four classification groups (European, African, Native American, and Asian), thus expanding the two-way classification of the OSSA score and including two additional population groups. Although the two methods are both based on the same approach, that is, the semi-quantitative assessment of almost the same morphoscopic traits, some disagreements between the methods were observed. For example, in some instances, the OSSA score and HefneR classified the same cranium into two different population groups (i.e., African/Black and European/White). With respect to these crania, it is noted that the probabilities provided by HefneR were distributed over more than one group, with the highest value equal to or below 0.65, except the case of cranium Sample 095 whose probability to be classified as African was 0.80. Again, the results can only be partially compared between methods because of the different classification groups: three cases were classified as Asian according to HefneR with probabilities lower than 0.8; however, this population group is not considered by the OSSA score.

**Figure 1:**
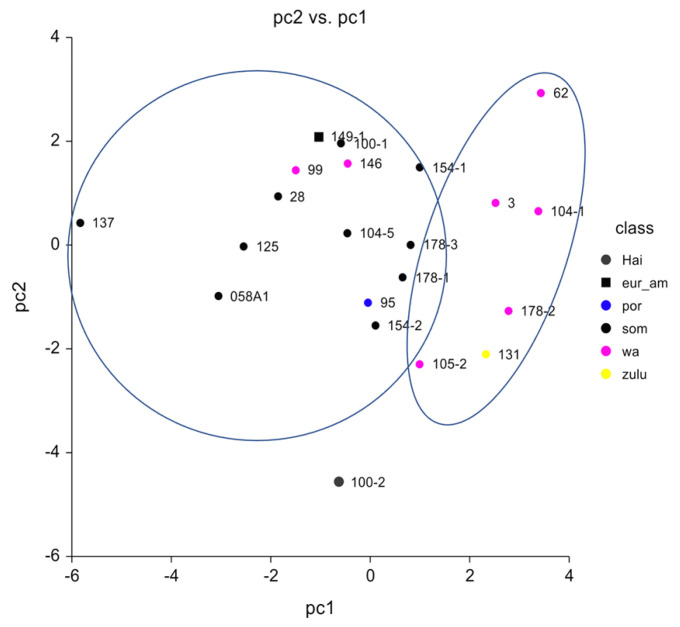
Principal coordinate (PC) plot of the craniometric analyses for ancestry estimation. The ellipses, fitted by eye, include the clusters closer to West Africa (right) and to Somali (left).

Based on the craniometric data, the population affinities are as follows: Somali 10, West African 7, Zulu 1, Portuguese 1, Euro-American 1, and Hainan 1. The posterior probabilities are generally low, only eight posterior probabilities are ≥0.7, five of which belong to Somali. Lower posterior probabilities are consequently distributed over several groups. Such a pattern indicates that low posterior probability crania do not fit well into any of the reference samples, which is to be expected given the likelihood that migrants come from populations other than those represented by the reference groups. Nevertheless, a strong African pattern emerges, 18 of 21 crania classify as either West African, Zulu, or Somali. The principal coordinate (PC) plot is shown in Figure 1. PC1 sorts those classified as Somali (PC1 scores <0) and from those classified as West Africa, including Zulu (PC1 scores >0). The ellipses, fitted by eye, include all or most of those with West African affinity vs. those with Somali affinity. The Somali ellipse includes two West Africans, the Euro-American, and the Portuguese. A *t*-test on the PC1 scores of Somali vs. West Africa and Zulu yields (t = 2.91, df = 16, *p* = 0.009). PC2 shows less dispersion and serves mainly to separate the cranium with Hainan affinity from the others.

The results are strongly suggestive of a substructure among these 21 crania. A course mesh can use the available samples to identify the substructure. Some groups that may have been on the ship are not represented, for example, Eritreans, and they predate the migrants by many decades. Future work may be able to more accurately identify a substructure.

For the morphological sex estimation, the results can be regrouped as follows:

1. Determined: When the morphoscopic methods agreed on the classification group (e.g., European and White), the sex classification followed the ancestry estimation, and therefore, the appropriate equations by Krüger et al. [11] for South African Black or South African White were applied. Eight crania belonged to this group: five crania displayed the highest probability to be classified as male for all the traits considered, whereas three crania were classified as female.
2. Uncertain: When the morphoscopic ancestry estimation provided consistent results between methods, but the cranium presented mixed traits and therefore mixed probabilities according to the equations for sex estimation. For eight crania, a definitive judgment could not be expressed, as the coexistence of feminine and masculine traits produced mixed probabilities from the equations: four crania were F/M and four were M/F.
3. Undetermined: When the morphoscopic ancestry estimation classified the cranium into different population groups, it was not possible to choose the appropriate re-calibrated equation by Krüger et al. [11]. Therefore, the result was based on Walker [7], since the method pools White and Black individuals. Five crania belonged to this group; the sex estimation based on Walker [7] classified two crania as female and three crania as male.

Cranial traits for sex estimation present inter-population variation [8]; thus, the published methods may demonstrate poor accuracy or even be unreliable when applied to samples other than those from which they were developed [11,12,44]. In the present study, the sex estimation methods calculate discriminant functions using equations that are based on combined traits (e.g., nuchal crest and mental eminence or mastoid process and glabella); thus, the coexistence of masculine and feminine traits in the same cranium can lead to mixed results, where the cranium is classified as female according to some traits and as male according to other traits. Apart from the cases where the ancestry was indeterminate due to inconsistent results between morphoscopic methods, some crania presented mixed traits (i.e., more feminine traits combined with more masculine traits), and therefore, the discriminant functions could not produce a unanimous result. Here, in nine cases that presented such a mixture, a conclusive estimation could not be expressed; therefore, the cranium was classified as M/F or F/M according to the higher number of equations that output a higher probability for the cranium to be male or female, respectively.

Following the craniometric analysis, the sex discriminant classified 7 of the 21 crania as female, although two had approximately equal posterior probabilities (i.e., cranium 104-1 and cranium 125), and therefore no firm conclusion could be drawn. The remaining five crania had relatively high posterior probabilities for female. Based on sex established by DNA, these are clearly misclassifications. What this tells us is that cranial morphology of some migrants is more gracile than the Zulu from which the sex discriminants were derived. Table 1 summarizes the results of the physical analyses.

A human cranium may present mixed traits that prevent an exact classification into single groups, both for ancestry and sex. As observed in this study, some cases could not be classified as male or female because they presented mixed probabilities according to different traits, and the sex/ancestral group into which a cranium was classified differed according to the method. The size of the sample was limited to 21 crania that were already genotyped for identification purposes; inter-individual variability of such fluctuating features for sex and ancestry estimation within this pool of crania may be therefore clarified by increasing the sample. When applying statistical methods for ancestry and sex estimation, anthropologists must consider that the reference population data may not be appropriate and representative of the sample of the study [5]. Another factor influencing the analyses may be related to secular change [6,13] potentially experienced by this population, which would benefit from further investigation. As a result, indefinite ancestry and sex estimations represent a possible scenario where the strong dependence of the methods on the original reference sample is the main element that may hamper the creation of a biological profile. 

### 3.2. Molecular Data

#### Sex Estimation and Skeletal Ancestry

DNA was extracted from the petrous portion of the temporal bone of 21 individuals, double-stranded DNA libraries were created, and a shotgun approach was used to determine the sex of each individual and to retrieve the complete mitochondrial genome, Y chromosome single nucleotide polymorphisms (SNPs), up to 597,573 SNPs across the human genome from each individual. In Table S1 (Supplementary Materials) the shotgun results of each sample are summarized.

Subsequently, sex estimation was determined by considering the ratio of sequences aligning to the X and Y chromosomes and the molecular analysis highlighted that all crania belonged to individuals of male sex. Regarding the ancestry estimation, 597,573 SNPs were selected across the human genome. The SNPs called for ancestry estimation of each individual are summarized in Table 2.

**Figure 2:**
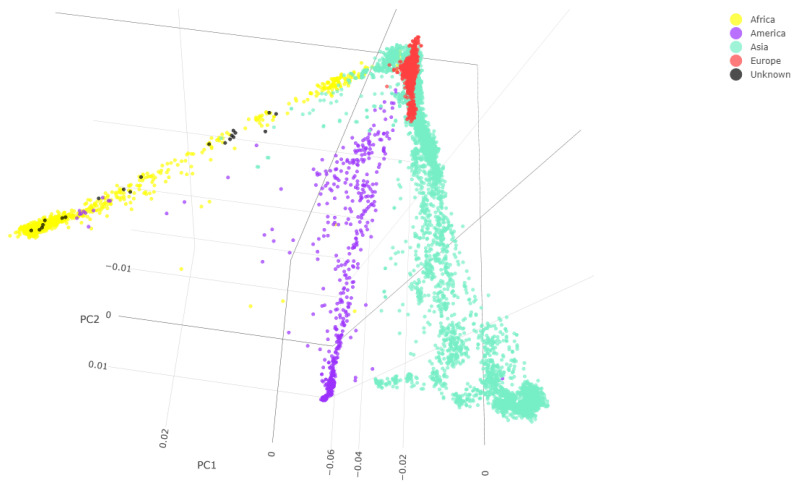
Individuals from the database belonging to the different continents are represented with different colors. Unknown individuals are in black. Score plots PC1, PC2, and PC3.

As can be observed in Table 2, the number of SNPs varies among different samples, ranging from 30,842 out of 597,573 SNPs for Sample 028 to 580,917 out of 597,573 SNPs for Sample 149. The principal component analysis plot (Figure 2) shows that our unknown samples form a genetic cluster that overlaps with the African individuals in the database. Therefore, the PCA analysis revealed relatedness/genetic similarity among all unknown individuals with the 1195 African individuals in the database.

**Figure 3:**
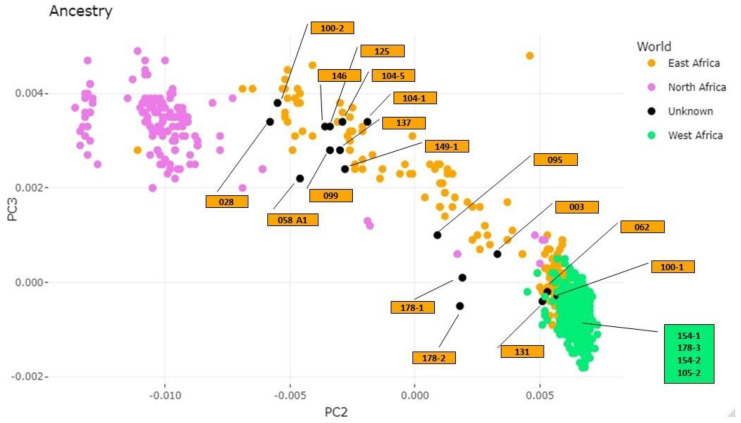
Individuals from the African database were subdivided into different geographical areas and each area is represented by a color. Unknown individuals are in black, and each black dot has been labeled with the specimen name. Score plots PC1, PC2, and PC3.

In an attempt to assess ancestry at the geographical area level, PCA was performed for the single African population after dividing it into three different areas (North Africa, East Africa, and West Africa). Individuals from Central and South Africa were not considered since the numbers of individuals in our datasets were low. It can be observed in Figure 3 that the African geographical areas form three different clusters and most of the unknown individuals (black dots in Figure 3) fall into the East Africa cluster, except for Samples 105-2, 154-1154-2, and 178-3 that fall into the West Africa cluster, as evaluated by comparison of the coordinates of database individuals and unknown samples.

In addition, to investigate the potential influence of sex biases in the biogeographical ancestry and to estimate when the common patrilineal and matrilineal ancestors lived, Y chromosome and mtDNA haplogroup were determined using, respectively, Yleaf and Mitomaster.

The Y chromosome results in terms of reads that mapped on the Y chromosome, number of SNPs called by Yleaf, QC scores, and haplogroup assigned by the software are reported in Table 3. An automatic prediction of the haplogroup with QC score greater than 0.81 was obtained for most samples. No prediction was shown for four samples (058 A1, 099, 100-1 and 104-5), probably because the QC score fell below 0.75. Therefore, manual interpretations of these samples’ specific output files were required, as suggested by the Yleaf manual (https://github.com/genid/Yleaf accessed on 22 December 2022).

As can be observed in Table 3, most of the male individuals analyzed belonged to macrohaplogroups E (approximately 66.7% of individuals) and J (19%), except for Sample 003 and Samples 099 and 104-5 that showed, respectively, macrohaplogroups B (4.8%) and A (9.5%). As presented in different papers (for example [45,46,47,48]), haplogroups A, B, and E are overwhelmingly the most common in the African population. Whereas haplogroup J shows high frequency in the Arabian Peninsula, southern Mesopotamia, and the southern Levant as proposed by Sahakyan et al. [49]; however, the same study also highlighted a moderate frequency of this haplogroup in North and East Africa. In addition, Wood et al. [46] found that haplogroup J was concentrated in about 20% of the Afro-Asiatic studied.

Y heatmap, a relative frequency map for Y haplogroups developed by Hunter Provyn and Thomas Krahn (https://phylogeographer.com/scripts/heatmap.php accessed on 30 December 2022), was used to show where most people with that specific haplogroup lived, or the ancestors who likely lived there. The results are summarized in Table 4.

Regarding the mitochondrial results, the number of reads that map on the mitochondrial genome, the number of bases covered, and the haplogroup assigned by MITOMASTER are summarized in Table 5.

As can be observed in Table 5, most of the individuals (90.47%) belong to haplogroup L, in particular to haplogroups L0 (5.26%), L2 (36.84%), L3 (52.63%), and L5 (5.26%), except for Samples 058 A1 and 104-5 that belong to, respectively, haplogroups T and U. The L haplogroups are African specific, indicating the African origin of our unknown individuals [50]. As proposed by Maier et al. [51] in their recent paper, the L0 subclade shows high frequency in South Africa and moderate frequency in Central and East Africa with a maximum distribution percentage (approximately 35%) of L0a (Sample 100-2) in Congo. Otherwise, the L2 subclade is distributed in most of Sub-Saharan Africa with a maximum distribution percentage (55%) of L2a (Samples 028, 131, 137, 146, 154-1, and 178-3) in Congo and with a distribution percentage of L2c (Sample 095) less than 19% in Mali [51]. The L3 subclade, instead, is distributed in most of Africa with low frequency in Botswana, South Africa and Algeria, Tunisia, and Libya. Figure S6 in the Supplementary Materials by Maier et al. [51] shows that the frequency of haplogroup L3b (Samples 062, 100-1, 178-1, and 178-2) has the greatest distribution percentage (approximately 18%) in Madagascar with high values also in Mali, Niger, and Chad. On the contrary, haplogroups L3e (Samples 003 and 105-2) and L3f (Sample 154-2) show, respectively, the greatest distribution percentage (48%) in the inner part of Western Sahara with high values also in Morocco and the greatest distribution percentage (11%) in Sudan. Haplogroups L3i (Samples 099 and 125) and L3x (Sample 149-1) show, respectively, the greatest distribution percentage (5%) in Somalia, Ethiopia, and Uganda and the maximum distribution percentage (7%) in Ethiopia. Finally, according to Maier et al. [51], haplogroup L5 (Sample 104-1) is distributed in most of Central Africa with the highest frequency in Congo (9.3%).

A different discussion must be made for Sample 058-A1 that belongs to haplogroup T1a. Haplogroup T makes up almost 10% of the mitochondrial genomes in Europe and approximately 8% in the Near East. Haplogroup T1 is distributed, albeit at varying frequencies, from Northwestern Africa throughout Europe, the Caucasus, and the Near East, into Western India, and across Central Asia into Siberia. The distribution of subclade T1a, despite being widespread, is nonhomogeneous and reaches values of approximately 4% and 8% also in Egypt and Algeria/Tunisia, respectively [52]. The high frequency of subclade T1a in Tunisia (38%) was also confirmed by Halim’s study [53]. Haplogroup U2 is mainly distributed in Western Eurasia [54] and, although rare, haplogroup U2d has also been found in Ethiopia in both the Amhara region and Tigray [55], presumably as a result of the subsequent penetration from Western Asia and the Caucasus due to the absence of haplogroup U2d in North Africa.

As with the Y haplogroups, the worldwide distribution of mitochondrial haplogroups was assessed using EMPOP (Table 6).

### 3.3. Comparison of Physical and Molecular Data

In such a complex scenario, it is obvious that the interaction of physical and molecular anthropologists is of paramount importance to magnify efforts towards reliable and sound biological profiles that may provide guidance on the sex and ancestral groups of victims, in order to direct the search for relatives. However, the times may not yet be mature for such a merger.

This work presented a multi-method study that investigated and discussed the possible difficulties that anthropologists confront when studying human crania of unknown sex and origin, within the context of a migrant population.

The physical morphological analyses indicate that the sample is mainly composed of males, of White/European ancestry, although in several instances a conclusive sex and ancestry estimation was not possible due to opposing or inconsistent results between methods. Craniometrics confirm that the crania are mainly male, although the population affinity is more frequently African (Somali and West Africa). This is in agreement with the molecular analyses that show that the crania exclusively belong to male, African individuals. Based on the identity cards that were found associated with some of the victims of the 18 April 2015 shipwreck (but not necessarily associated with these crania), it seems likely that this ship carried people from Sub-Saharan countries. Clearly, no documents were found with the disarticulated crania; therefore, this is a completely unknown sample with respect to sex and ancestry. This hinders the evaluation of the accuracy of the estimations. However, this work did not aim to compare the accuracy rates of anthropological methods against DNA typing, but aimed to stress the elemental interdisciplinary teamwork of physical and molecular anthropologists and the difficulties that may emerge when dealing with biological profiles especially of migrant populations. Skeletal sex and ancestry estimation results based only on morphological traits of the cranium have been found to be 92% accurate compared to the DNA results [56]. Accuracy rates on skeletal ancestry (versus ancestry determined by documents) have been at 90% [57]. Here, it is not possible to assess the reliability of the observations because there is no positive or negative control sample, given the completely unknown origin of the sample, which represents both the major shortcoming and the purpose of this study. The contrasting physical results with respect to ancestry somehow also affect the sex estimation of these crania, since this is also usually inferred according to the ancestral group of the individual [58]. These crania were, therefore, considered to be “undetermined” both for ancestry and sex following Thomas et al. [57]. It should be pointed out that seven crania did not show complete skeletal and/or dental development. This may divert a conclusive sex and ancestry estimation by physical means given that related characteristics may be undeveloped [59], thus further reducing the reliability of the observations. Therefore, molecular analyses may prove to be essential to infer at least the genetic sex in individuals that present such an ambiguous set of traits and prevent a robust estimation [60].

Novel specific formulae that are more accurate for a given population can be suggested and tested for their validity, proving their reliability when applied to the appropriate sample [12]. However, the ancestry and sex prediction parameters, and especially the methods that strive for a quantification of the results, strongly depend upon the reference population on which they are developed, thus limiting the application to other populations [61]. This study raised the following question: How can anthropologists tackle the issue of sex and ancestry estimation when dealing with unknown specimens such as those of this study? With respect to sex, although this study lacks a positive control that could derive from the personal identification of these victims, the molecular analyses can be considered to be more reliable due to the stability of the genetic code and its determination. Regarding ancestry, this work shows that anthropology still finds itself mired in an endless conundrum: Which reference population is appropriate to estimate the ancestry of individuals? As morphology classifies crania into macro groups, craniometric analyses seem to provide more exhaustive information. Therefore, decision support systems and methods enhanced by statistical frameworks can provide valuable insights for physical analyses, but only by guiding anthropologists through the interpretation of evidence and decision making rather than by identifying binding groups [8]. Regarding the molecular analysis, the 597,573 SNP callings in association with the PCA model were assessed to infer the biogeographical ancestry of unknown individuals. The PCA analysis (also known as exploratory analysis), which is one of the most used statistical clustering methods for the inference of the biogeographical ancestry of a person, allowed us to create four separate clusters corresponding to African, American, Asian, and European individuals and unequivocally assign all the 21 crania to the African population through a visual, intuitive, and easy-to-interpret approach. The autosomal data were also confirmed by the analysis of uniparental markers (mtDNA and Y chromosome) and the assignment of the African haplogroup to all 21 analyzed crania. In addition, athe PCA was also performed at an intra-continental level considering only the African population and dividing it into three different areas (North, West, and East Africa). The obtained results highlight that most of the unknown individuals fall into the East Africa cluster, except for Samples 105-2, 154-1154-2, and 178-3 that fall into West Africa. However, since PCA modeling is not a reliable discrimination/classification model [62] and the individuals from Central and South Africa were missing from the database used, the ancestry inference, especially at an intra-continental level, may not be accurate. The difficulty of ancestry inference at an intra-continental level was also confirmed by the results of uniparental markers. In summary, the molecular analysis clearly inferred the continent of origin of the 21 crania, but it was not possible to define a single geographical area of origin for each cranium. In these cases, particular attention should be paid to the database. Since the analysis of ancestry inference was performed by comparing the sample genotype with one or more known reference population groups, well-characterized databases with high-quality genotyping results of well-defined reference populations are critical.

**Figure 4:**
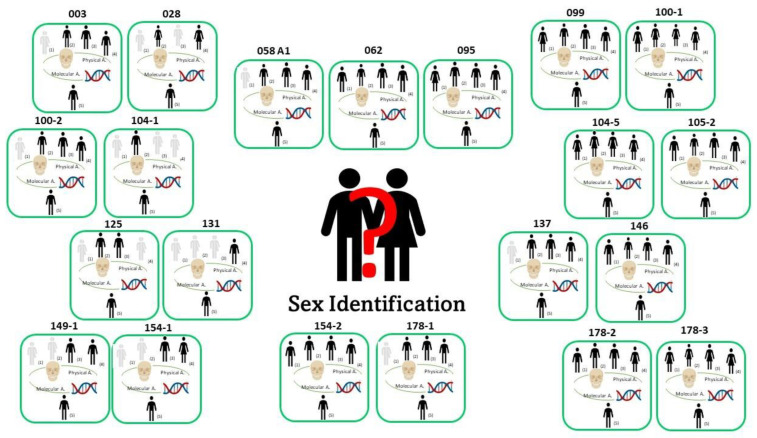
Physical and molecular sex estimation results: (1) South African White [11]; (2) South African Black [11]; (3) Walker [7]; (4) craniometrics; (5) molecular method [37]. 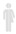, male/female and female/male; 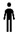, male; 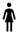, female.

**Figure 5:**
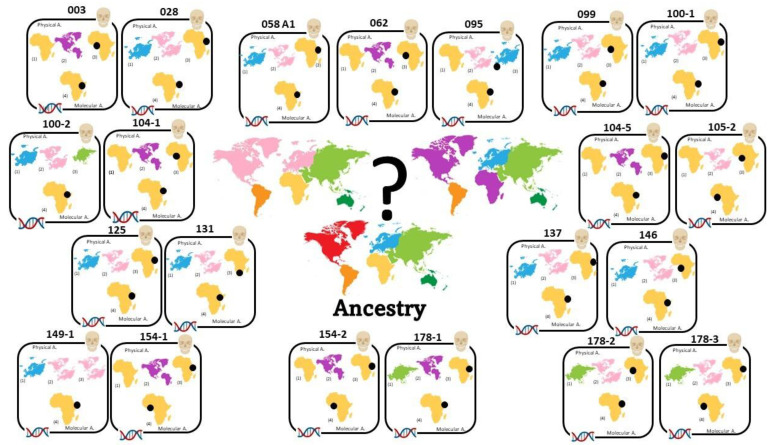
Physical and molecular ancestry estimation results: (1) HefnerR: African, European, Asian, Native American [24]; (2) OSSA score: Black (Afro-American), White (Euro-American) [23]; (3) Craniometrics: Somali, Zulu, West Africa, Portuguese; Euro-American; Hainan (Asia); (4) SmartPCA [38]. 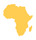, African; 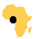, West African; 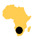, Zulu; 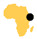, Somali; 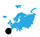, Portuguese; 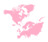, Euro-American; 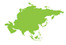, Hainan (Asia); 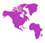, Afro-American.

Despite this, as shown in Figure 4 and Figure 5 where the comparison between anthropological (physical and molecular) results relating to sex and ancestry are summarized, the molecular analysis support the physical data in both sex and ancestry inference, confirming the presence of 21 male crania of African origin. As proposed by Alladio et al. [62] and further developed by Pilli et al. [63], to infer intra-continental biogeographical ancestry in a forensic context, we suggest adopting a well-characterized and well-defined database and a robust classification method such as a multivariate statistical and machine learning approach associated with selected markers (AIMSNPs ancestry informative markers).

Again, our results cannot not be supported by antemortem data, but this was not the scope of the project. On a final note, physical and molecular analyses can produce contrasting results where an anthropologist is not able to confidently determine sex and ancestry, thus preventing the elaboration of the biological profiles and the identification activities. Although a strict reliance on reference groups still represents a drawback when analyzing completely unknown specimens, an approach that intertwines physical and molecular anthropology may start breaking through this everlasting issue with further comparative studies. As such, the expectation of the authors is that investigation of this research avenue would pave the way to the development of novel tools, methods, and reference databases that may help to overcome the limitations of both disciplines.

## 4. Conclusions

This paper presents the anthropological and genetic analyses for sex and ancestry estimation of 21 disarticulated crania that belong to unidentified migrants who perished in the Mediterranean shipwreck of 18 April 2015. This study highlights the drawbacks that challenge physical and molecular anthropologists when presented with such a peculiar set of remains. In some cases, physical and molecular analyses produced differing results (for sex estimation 8 out of 21 crania showed discrepant results, while for ancestry estimation 3 or 12 out of 21 crania showed discrepant results, according to the method) highlighting that the methods were still strictly linked to reference populations. Within the context of a migrant population, the application of a multimethod approach that combines molecular and physical evidence may provide valuable insights for elaborating the biological profiles of unidentified crania. However, according to the authors’ experiences, a method to merge the two disciplines and to reach a univocal and conclusive result is still to be fully explored.

## Figures and Tables

**Table 2:** A summary of ID samples, SNPs called for ancestry estimation of each individual, and the PCA results.

ID Samples	ASNPs 597573	PCA Result
003	51,100	Africa
028	30,842	Africa
058 A1	163,213	Africa
062	406,751	Africa
095	128,947	Africa
099	197,047	Africa
100-1	184,076	Africa
100-2	124,672	Africa
104-1	140,034	Africa
104-5	89,217	Africa
105-2	205,032	Africa
125	177,666	Africa
131	561,124	Africa
137	147,779	Africa
146	224,868	Africa
149-1	580,917	Africa
154-1	139,467	Africa
154-2	164,138	Africa
178-1	165,037	Africa
178-2	66,289	Africa
178-3	188,702	Africa

**Table 3:** ID sample, number of reads mapped on the Y chromosome, number of SNPs used for the Yleaf analysis of each individual, and QC score, i.e., an overall quality score of the predicted haplogroup. The haplogroups that were assigned manually are shown in red.

ID Sample	Number of Reads (ChrY)	SNP (Yleaf)	Haplogroup (Yleaf)	QC Scores
003	15,483	2498	B2a1a1a1	1.0
028	9467	1363	J1a2a1a	1.0
058 A1	581,646	9380	E1b1b1b2a1a1a1a	0.0
062	672,312	26,382	E1a2a	0.846
095	38,448	5903	E1b1b1b1b1a	1.0
099	47,585	9922	A1b1b2b2~	0.0
100-1	54,206	8700	E1b1a1a1a1c2c3a1b	0.0
100-2	33,706	5793	E1b1b1a1a1b1a	0.99
104-1	40,152	6489	J1a2a1a2d2b2b2c4d2a2a5a1c	0.992
104-5	21,882	3849	A1b1b2b~	0.0
105-2	62,940	9998	E1b1a1a1a1c1b2a	1.0
125	51,004	8626	J1a2a1a1a	1.0
131	1,458,353	46,817	E1b1a1a1a1c2~	0.956
137	39,324	7032	E1b1b1b2a1a1a1a1a~	1.0
146	68,090	11,144	J1a2a1a2d2b2b2c4d1a1a1	0.971
149-1	2,013,574	52,816	E1b1b1a1a1b1	0.991
154-1	38,776	6300	E1b1a1a1a1c2c3a2a	1.0
154-2	47,774	7709	E1b1a1a1a1c1b	1.0
178-1	49,253	7946	E1a2b1a2	0.815
178-2	36,180	3093	E1a2b1a2	0.95
178-3	55,083	8982	E1b1a1a1a1c2	1.0

**Table 4:** ID sample, haplogroup using Y full nomenclature, world distribution of haplogroups observed by heatmaps. Purple, orange, and light-yellow colors refer to greater, intermediate, and less presence of that specific haplogroup, respectively.

	World Distribution of Y Full Heatmap			
ID Sample	Haplogroup Yfull	Purple Zone	Orange Zone	Light Yellow Zone
003	B-M5844	Saudi Arabia	Kuwait	United States; Jordan; Israel; Qatar; United Arab Emirates; Egypt; Sudan; Chad; Central African Rep.; Cameroon; Kenya; South African
028	J1	Saudi Arabia		Most of America, Asia and Europe; North-East Africa and Oceania
058 A1	E-P147	Saudi Arabia	United States; Yemen; Algeria; Gambia; Nigeria; Italy; Kuwait; United Arab Emirates; Albania; United Kingdom	Asia; Europe; Most of Africa; Canada; Most of Latin America
062	E-CTS736	Nigeria; Saudi Arabia		
095	E-Y141678	Morocco	Jordan	Mali
099	A-Y24713	Saudi Arabia	Ethiopia	Yemen
100-1	E-Z6015	Gambia	Spain	Morocco; Sierra Leone
100-2	E-CTS2294	Somalia	Chad; Ethiopia; Kenya	Cameroon; Sudan; Egypt; Libya; Eritrea; Yemen; Iraq; Jordan
104-1	J-P56	Saudi Arabia	Yemen; Ethiopia	Eritrea; Egypt; Iran; Kuwait; Bahrain; United Arab Emirates
104-5	A-Y23655	Saudi Arabia	Ethiopia	Yemen; Sudan
105-2	E-FT212537	Saudi Arabia		
125	J-P56	Saudi Arabia	Yemen; Ethiopia	Eritrea; Egypt; Iran; Kuwait; Bahrain; United Arab Emirates
131	E-CTS9883	Gambia	Sierra Leone; Senegal	Guinea; Algeria; Morocco; Egypt; Saudi Arabia; Spain
137	E-Y160200	Yemen	Egypt; Saudi Arabia; Oman	
146	J-Z18257	Yemen	Saudi Arabia	Algeria
149-1	E-Y205079	Saudi Arabia; Ethiopia		
154-1	E-Z6018	Gambia		
154-2	E-L515	United States; Sierra Leone	Nigeria; Saudi Arabia	Morocco; Niger; Burkina Faso; Ghana; Cameroon; United Kingdom
178-1	E-Z5987	Gambia		
178-2	E-Z5987	Gambia		
178-3	E-CTS9883	Gambia	Sierra Leone	United States; Mexico; Senegal; Guinea; Morocco; Algeria; Egypt; Saudi Arabia; Spain

**Table 5:** ID sample, number of reads on mitochondrial DNA, and haplogroup assigned by the Mitomaster software.

ID Sample	Number of Reads (MT)	Haplogroup (Mitomaster)
003	5054	L3e (L3b1b)
028	4167	L2a (L2a1+143+16189 (16192))
058 A1	51,729	T1a
062	171,521	L3b (L3b1a+@16124)
095	12,327	L2c (L2c)
099	11,837	L3i
100-1	12,939	L3b (L3b1a)
100-2	10,057	L0a (L0a1a+200)
104-1	15,459	L5b (L5b1)
104-5	9498	U2d (U2d)
105-2	14,120	L3e (L3e2a)
125	14,930	L3i (L3i2)
131	285,062	L2a (L2a1c)
137	9793	L2a (L2a1j)
146	19,302	L2a (L2a1c)
149-1	492,088	L3x (L3 × 1b)
154-1	11,931	L2a (L2a1c)
154-2	15,427	L3f (L3f1b4a)
178-1	11,802	L3b (L3b1a+@16124)
178-2	7010	L3b (L3b1a+@16124)
178-3	13,914	L2a1a1

**Table 6:** Sample ID, haplogroup according to EMPOP nomenclature, worldwide distribution of haplogroups observed from heatmaps. Red zone refers to a major presence of the haplogroup, orange to an intermediate presence, yellow to a minor presence, and blue to a very low presence.

	World Distribution of EMPOP Heatmap				
ID Sample	Haplogroup EMPOP	Red Zone	Orange Zone	Yellow Zone	Blue Zone
003	L3b1b	Morocco			United States
028	La2a1+143+16189 (16192)	Somalia	United States	Morocco; Senegal; Gambia; Sierra Leone; Liberia; Burkina Faso; Ghana; Togo; Benin	United States
058 A1	T1a	Europe	Middle East	United States	United States
062	(L3b1a+@16124)	United States; Spain; Portugal		Morocco; Ghana; Togo; Benin	Middle East; Egypt; Brazil; Venezuela
095	L2c	United States; Spain; Portugal; Senegal; Gambia	Morocco; Guinea; Sierra Leone; Cote d’Ivoire; Ghana; Togo	Brazil	United States
099	L3i1a	Somalia; Uganda; Middle East;			
100-1	L3b	United States; Spain; Portugal; Morocco; Cote d’Ivoire; Burkina Faso; Ghana; Togo; Kenya; Uganda	Senegal; Gambia; Guinea	Cuba; Brazil	United Stated; Middle East; Egypt
100-2	(L0a1a+200)	United States (Los Angeles)		Most of United States; Egypt; Senegal; Gambia; Somalia; Kenya	Part of the United States; Brazil; Nigeria; Cameroon; Gabon; Middle East
104-1	(L5b1)	Uganda			Egypt
104-5	(U2d)	Portugal; Spain; Southern Europe; Turkey; Syria			Middle East; United States; Argentina; Uruguay
105-2	L3e2	United States; Brazil; Portugal; Spain; Cote d’Ivoire; Ghana; Togo; Benin			Middle East; Kenya; Uganda; Nigeria; Cameroon; Gabon
125	(L3i2)	Somalia			United States; Middle East; Uganda; Kenya
131	L2a1+143	United States; Cote d’Ivoire; Ghana; Togo; Benin; Somalia	Portugal; Spain; Morocco	Brazil; Uganda; Little part of the Middle East	Egypt; Middle East; Southern Europe
137	L2a1+144	United States; Cote d’Ivoire; Ghana; Togo; Benin; Somalia	Portugal; Spain; Morocco; Senegal; Gambia;	Brazil; Uganda; Little part of the Middle East; Brazil	Egypt; Middle East; Southern Europe
146	L2a1	United States; Cote d’Ivoire; Ghana; Togo; Benin; Portugal; Spain	Brazil	Senegal; Gambia; Guinea; Little part of the Middle East; Somalia	Egypt; Middle East; Tunisia; Uganda; Kenya; Southern Europe
149-1	L3x (L3x1b)	Somalia		Saudi Arabia	Little part of the Middle East
154-1	L2a1+143	United States; Cote d’Ivoire; Ghana; Togo; Benin; Somalia	Portugal; Spain; Morocco;	Senegal; Gambia; Guinea; Brazil; Uganda; Little part of the Middle East; Brazil	Middle East; Southern Europe; Egypt
154-2	L3f1b4	Brazil	United States; Portugal; Spain	Gabon	Cameroon; Nigeria; Cote d’Ivoire; Burkina Faso; Ghana; Togo; Benin; Uganda; Kenya; United Arab Emirates; Little Part of Europe
178-1	(L3b1a+@16124)	United States; Portugal; Spain		Ghana; Togo; Benin; Morocco	Brazil; Nigeria; Egypt; Middle East
178-2	(L3b1a+@16124)	United States; Portugal; Spain		Ghana; Togo; Benin; Morocco	Brazil; Nigeria; Egypt; Middle East
178-3	L2a1a2	United States	Portugal	Gabon	Cameroon; Nigeria; Cote d’Ivoire; Burkina Faso; Ghana; Togo; Benin; Uganda; Kenya;

## Data Availability

The data presented in this study are available upon request.

## Supplementary Materials

The following supporting information can be downloaded at: https://www.mdpi.com/article/10.3390/genes14030706/s1, Table S1: Eager report.
